# IL‐12 and IFN‐γ in Response to *Leishmania Infantum* Antigens in Felines From an Endemic Area for Visceral Leishmaniasis

**DOI:** 10.1111/pim.70021

**Published:** 2025-08-12

**Authors:** João Victor Lé Lode, Lucas Takeshi Siqueira Ito, Gisele Mitsue Umino, Valéria Marçal Felix de Lima

**Affiliations:** ^1^ Department of Clinical Medicine, Surgery and Animal Reproduction São Paulo State University (UNESP), School of Veterinary Medicine Araçatuba São Paulo Brazil

**Keywords:** Assymptomatics, ELISA, feline, IFN‐γ, IL‐12, leishmaniasis, parasite load

## Abstract

Visceral leishmaniasis is a potentially fatal zoonosis with an increasing incidence. Most infected felines present the disease in its subclinical form and demonstrate greater resistance to parasitemia than dogs. However, the role of cellular immunity in felines is still poorly understood. This study measured serum levels of interleukin (IL)‐12 and interferon‐gamma (IFN‐γ) in asymptomatic cats naturally infected with antibodies against *Leishmania* spp. and in uninfected cats. IL‐12 and IFN‐γ were measured in serum samples by ELISA. Parasite load quantification was performed on DNA from bone marrow samples using qPCR. Cats naturally infected by *Leishmania spp*. showed significantly higher serum levels of IL‐12 and IFN‐γ compared to control animals. IL‐12 showed a positive correlation with IFN‐γ suggesting a regulatory role of IL‐12 in activating the Th1 immune response, which enhances macrophage function and promotes intracellular parasite elimination. Additionally, IL‐12 showed a moderate negative correlation with parasite load, indicating a protective effect of IL‐12 in feline leishmaniasis. These findings suggest that IL‐12 and IFN‐γ play critical roles in modulating the feline immune response against parasitic infection, possibly contributing to the control of parasite replication and in the prevention of clinical signs. The immune response observed in felines could be explored for future immunotherapeutic approaches, helping to mitigate the progression of leishmaniasis in cats and reducing the risk of transmission in endemic regions.

**Summary**

- Felines infected with Leishmania spp. presented higher serum levels of IL‐12 and IFN‐γ.
- There is a positive correlation of IL‐12 and IFN‐γ.
- There is a negative correlation of IL‐12 and the load of Leishmania spp. in felines.
- High IL‐12 and IFN‐γ levels are associated with asymptomatic disease in felines.

## 1 Introduction

Visceral leishmaniasis (VL) is a zoonosis with a worldwide impact, considered to be an obligate intracellular parasitic disease caused by a protozoan of the genus Leishmania, with *Leishmania (L.) infantum* (synonymous with *L. chagasi*) being the main etiological agent in Brazil. Its transmission occurs after the female mosquito of the genus Lutzomyia, belonging to the *Phlebotominae* family, hematophagously inoculates the parasite, in its promastigote form, into the definitive host [1]. Human VL is fatal if left untreated in over 95% of cases. Most cases occur in Brazil, East Africa, and India [2].

Domestic dogs ( *Canis lupus familiaris* ) are considered the main reservoirs of the protozoan in urban and peri‐urban areas [1]; domestic cats ( *Felis catus* ) can also act as reservoirs of the disease, although they are underdiagnosed, making them important targets for investigating pathogenesis [2]. However, the role of cats in maintaining transmission of the parasite is still uncertain [3]. Urban verticalisation, changes in lifestyle, and the demand for pets with more independent habits suited to smaller spaces have brought cats closer to humans, playing a fundamental role in the amplification of the peridomestic cycle of leishmaniasis, increasing the attention given to their health and basic care [4].

Feline Visceral Leishmaniasis (FelVL) is considered emerging and is frequently reported in countries such as the Middle East, Europe [5, 6, 7] and South America, especially in Brazil [8, 9], with sporadic reports in non‐endemic locations [10]. To date, six species of the *Leishmania* genus have been described in cats: *L. amazonenis, L. braziliensis, L. infantum, L. major, L. mexicana*, and *L. tropica* [3]. A major contributor to the spread of leishmaniosis to non‐endemic areas is globalisation. The movement of companion animals is a relevant factor in the spread of infectious agents, representing an epidemiological risk both for animals transported to endemic areas and for the introduction of pathogens into regions previously free of infection. This scenario can directly impact animal health and pose a threat to public health [11].

*L. infantum* infection in cats is generally not associated with the presence of classic clinical signs of the disease, remaining subclinical [12]. It can be explained by the difference in the immune response to *Leishmania* between dogs and cats [13], which plays a crucial role in controlling the infection. Compared to dogs, cats exhibit a degree of resistance to *Leishmania*, which results in a lower prevalence of infection and the development of clinical manifestations [14]. In the clinical phase, cats commonly present cutaneous, ophthalmic, and oral lesions, lymphadenopathy, weight loss, anorexia, and lethargy [2, 11]. These animals are frequently diagnosed with co‐infections or immunosuppressive comorbidities, most notably feline leukaemia virus (FeLV) and feline immunodeficiency virus (FIV), which may contribute to disease progression by impairing host immune responses [12].

In addition, the cytokines present in the microenvironment determine the immune response triggered [6]. It is known that in Canine Visceral Leishmaniasis (CVL) [15] and murine models of leishmaniasis [16], T cells produce cytokines that activate the microbicidal action of macrophages, resulting in the elimination of the parasite. The adaptive response therefore begins with the presentation of the *L. infantum* antigen by professional antigen‐presenting cells such as macrophages and dendritic cells to CD4+ T cells, which modulate the type of immune response to be established [15, 16].

IL‐12 is essential for the development of the T helper 1 (Th1) cellular immune response, characterised by the production of IFN‐γ, IL‐2 and tumour necrosis factor alpha (TNF‐α) [17, 18]. These cytokines stimulate phagocytosis by macrophages and their activation, leading to the production of nitric oxide (NO) and reactive oxygen species, promoting the elimination of the intracellular parasite [19]. This type of response has been associated with resistance to *L. infantum* infection in murine models [20, 21] and dogs [19]. However, the T helper 2 (Th2) humoral response predominates, with exacerbated antibody production [22] and the production of anti‐inflammatory cytokines (IL‐4, IL‐10, TGF‐β), increasing the survival of the parasite and leading to the appearance of the symptomatic picture of the canine disease [23]. In addition, increased levels of IL‐10 and factor growth tumour beta (TGF‐β) in human mononuclear cells suppress NO production, reducing the microbicidal activity of macrophages [24]. The immune response to VL in other models has been previously characterised and is crucial for understanding the Th1/Th2 dichotomy. However, the modulation of the immune response in cats during infection with *L. infantum* has yet to be elucidated.

In cats, the production of IFN‐γ is induced upon stimulation of blood cells with soluble *Leishmania* antigen, indicating the activation of a predominantly cellular immune response against the parasite [25]. Similar production of IFN‐γ has also been observed in response to antigen stimulation of whole blood or peripheral blood mononuclear cells (PBMCs) from cats exposed to other pathogens, such as *Feline coronavirus* [26] and *Toxoplasma gondii* [27]. The effect of the cytokine IL‐12 on the replication and apoptosis of FEL‐039 cells infected with FIV in vitro has been investigated, revealing an increase in IFN‐γ expression following the addition of IL‐12 to the cell culture medium. These findings suggest a pivotal role for IL‐12 in modulating the immune response [28]. Although these findings underscore the potential involvement of IL‐12 in immune regulation, the role of this cytokine in cats with leishmaniasis remains unexplored.

Despite the increase in cases of FelVL, few studies have evaluated the cellular immune response of these animals [25]. Therefore, this study quantified the cytokines IL‐12 and IFN‐γ, cytokines involved in cellular immunity, in the blood serum of asymptomatic felines with FelVL and compared them with those of healthy uninfected felines, also correlating IL‐12 with parasite load and serum IFN‐γ levels.

## 2 Methods

### 2.1 Ethics

All the procedures and methods used in the study were submitted to and approved by the Ethics Committee on the Use of Animals—CEUA of the Araçatuba School of Dentistry—UNESP, Araçatuba, SP (CEUA Process no.: 212/2023).

### 2.2 Screening the Animals

The animals' whole blood and bone marrow samples came from felines who were candidates for elective orchiectomy or ovariohysterectomy at the Zoonosis Control Center of Araçatuba–SP (CCZ), after the owners had signed a consent form. All cats were clinically evaluated by the attending veterinarian through a general physical examination, which included inspection for possible skin lesions, assessment of body condition, rectal temperature, mucous membrane colour, skin turgor, palpation of the popliteal and submandibular lymph nodes, and cardiopulmonary auscultation to detect possible changes consistent with infectious diseases or FelLV.

The *Leishmania*‐infected group consisted of 21 cats of various ages, breeds, and both sexes. These animals showed no characteristic signs of FeLV, as described by Pennisi et al. (2015) and no clinical findings suggestive of other infectious diseases. Feline Leishmaniasis diagnosis was performed using an indirect ELISA serological test, following an adapted protocol from Lima et al., 2005 and by qPCR to detect *Leishmania* spp. DNA in bone marrow samples. In addition, all selected cats underwent serological testing to assess the presence of FeLV antigen and antibodies against FIV to determine possible coinfections.

In the healthy feline group, we selected 10 cats of varying breeds, ages, and sexes that were clinically healthy. All cats tested negative by indirect ELISA serology and qPCR for FelLV.

### 2.3 Sample Collection and Storage

Blood and bone marrow were collected after a dissociative anaesthetic procedure using tiletamine hydrochloride and zolazepam (Telazol) 5 mg/kg and acepromazine 0.01 mg/kg intramuscularly.

Whole blood was collected by puncturing the jugular vein of the cats in both groups. One millilitre (mL) was placed in a plastic tube containing K2EDTA (Becton‐Dickson, USA) for DNA extraction, and 2 mL were placed in a plastic tube containing clot activator (Vacuplast GmbH, Austria) to obtain blood serum, which was separated by centrifugation at 3000 rpm for 5 min. Finally, the samples were stored in an ultrafreezer at −80°C for 4 months for subsequent dosing of cytokines and ELISA serological testing.

Bone marrow was collected by intraosseous puncture of the proximal region of the humerus or iliac crest using a hypodermic needle after clipping and antisepsis of the area. The sample was placed in a plastic tube containing K2EDTA (Becton‐Dickson, USA) and stored at −20°C for DNA extraction.

### 2.4 ELISA for Anti‐*Leishmania* spp. IgG Antibodies

Anti‐*Leishmania* spp. antibodies were tested using an enzyme‐linked immunosorbent assay (ELISA). High‐binding plates with a half‐area cavity (Greiner Bio‐One Microlon 600, Germany) were coated with 50 μl/well of 20 μg/ml crude antigen extract obtained from the culture of *L. chagasi* promastigotes MHOM/BR00/MERO2 [29] in carbonate/bicarbonate buffer (0.05 M, pH 9.6). After incubation at 4°C overnight, the following day the plates were washed three times with PBS (pH 7.0) and 0.05% Tween 20 (wash buffer) and blocked with 100 μl/well of PBS containing 10% fetal bovine serum for 1 h at room temperature and washed three times again. The serum samples (50 μL) were diluted 1:100 in PBS containing 10% fetal bovine serum and 0.05% Tween 20 and added to each well, in duplicate, then incubated for 3 h at room temperature. The positive control used came from a symptomatic animal, positive in the parasitological examination of lymph node puncture (gold method) and from Andradina–SP, Brazil; the negative control was an animal from a non‐endemic area (São Vicente‐SP, Brazil). After three washes with PBS containing 0.05% Tween 20, 50 μl/well of feline anti‐IgG conjugate (Anti‐cat IgG Peroxidase Conjugate SAB3700059 Sigma, St. Louis, USA) at a dilution of 1:500 was added and incubated for 1 h at room temperature. After three normal washes with the same solution, 50 μl/well of substrate solution was added, containing 0.4 mg/mL of O‐phenylenediamine (Sigma) and 1.6 L/mL of H_2_O_2_ in phosphate–citrate buffer (pH 5.0).

The reaction was stopped by adding 50 μl of 16% HCl. The optical density (OD) was measured using a Spectra CountTM reader (Packard Bio Science Company, USA) at a wavelength of 490 nm. The cut‐off point was established based on the sensitivity and specificity analysis using the ROC curve (receiver operating characteristic) of the individuals from the area not endemic for VL and from the area endemic for VL, who obtained the highest titers within the group evaluated. Animals with an O.D. above 0.350 were considered positive.

### 2.5 DNA Extraction and Quantification of the Parasite Load of *Leishmania* spp.

DNA from bone marrow and blood was extracted using the phenol‐chloroform protocol (Sambrook et al. 1989) and analysed using a NanoDrop ND‐1000 spectrophotometer (NanoDrop, Thermo Fisher, MA, USA) for purity (260/280) and concentration (ng/μL). After extraction, it was stored at −20°C until analysis. The β‐actin gene was used as a reference control for assessing DNA quality.

To quantify the parasite load of *L. infantum*, qPCR was carried out using primers targeting the parasite's circular kinetoplast DNA (kDNA) (forward: 5′‐GTGGGGGGAGGGGCGTTCT‐3′ and reverse: 5′‐ATTTTACACCAACCCCCAGTT‐3′). The qPCR reaction was standardised with 10 ng of purified genomic DNA, 4 μL of 5× HOT FIREPol Evagreen qPCR Supermix (Solis BioDyne, Tartu, Estonia), 0.5 μL of each primer and 13 μL of ultrapure H2O, in a final reaction volume of 20 μL.

A linear standard curve with 6 logarithmic ranges of concentration points was also generated using DNA extracted from *L. infantum* promastigotes (MHOM/BR00/MER02). The reactions were carried out on the Mastercycler RealPlex2 system (Eppendorf, CT, USA), under the following conditions: initial heating to 95°C for 12 min, followed by 40 cycles of denaturation (95°C for 15 s), annealing (65°C for 20 s) and extension (72°C for 20 s). After these steps, a dissociation curve of the amplified fragment was determined (95°C for 15 s, then 60°C to 95°C in 15 s/°C). The parasite DNA load was determined and samples with a threshold cycle and melting temperature corresponding to the standard curve were considered positive.

### 2.6 Immunochromatography for FeLV and FIV

The animals that screened positive for *Leishmania* spp. by ELISA or qPCR were also evaluated for coinfection with retroviruses. For this purpose, the presence of the FeLV p27 antigen and IgG antibodies against FIV were tested in the serum samples via rapid tests carried out with an ELISA kit (SNAP FIV/FeLV Antigen Combo Test: IDEXX Laboratories, Westbrook, ME, USA). The test was performed and interpreted following the manufacturer's guidelines.

### 2.7 Capture ELISA for the Detection of IL‐12 and IFN‐γ

Quantification of the cytokine IL‐12 and IFN‐γ in the serum of cats with leishmaniasis and healthy cats was carried out using the commercial Feline IL‐12p40 ELISA kit (RayBiotech, USA) and Feline IFN‐ γ ELISA kit (Invitrogen, USA) following the manufacturer's instructions.

### 2.8 Statistical Analysis

Statistical analysis was carried out using GraphPad Prisma V6 software (GraphPad Software Inc., La Jolla, CA, USA). The variables were tested for normality using the Shapiro–Wilk test. To compare the groups, the Mann–Whitney test was used for the IL‐12 and IFN‐γ variables. Spearman's test was used to correlate serum IL‐12 levels with parasite load in the leishmaniasis group. Pearson's test was used for the correlation between serum IgG anti‐*Leishmania* antibodies and *Leishmania* spp. DNA, and IL‐12 levels and IFN‐γ in the leishmaniasis group. The values were considered significant in the analyses when *p* < 0.05.

## 3 Results

### 3.1 Clinical and Laboratory Findings

**FIGURE 1:**
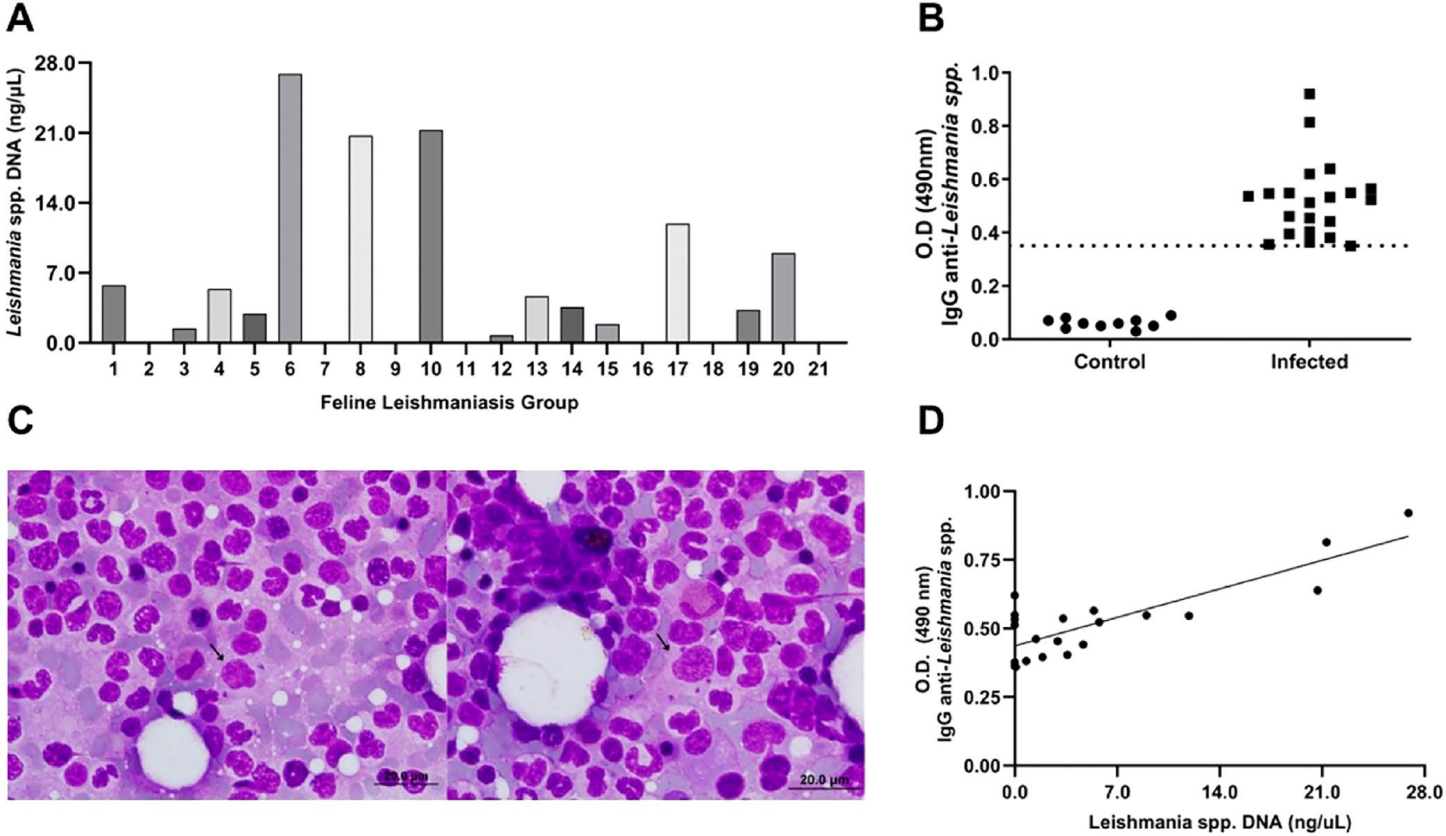
Quantification of *Leishmania* spp. DNA (qPCR) in the bone marrow of cats (*n* = 21) diagnosed with leishmaniasis and seropositive for *L. infantum* antigens by indirect ELISA (A). IgG reactivity to total *L. infantum* antigen in feline serum samples (*n* = 31) assessed by indirect ELISA (B). Serum IgG reactivity against total *L. infantum* antigen in healthy cats from non‐endemic areas (negative control, *n* = 10) and in seropositive cats (positive, *n* = 21). Representative images of bone marrow smear (Diff‐Quick stain 100×) from cats with leishmaniasis showing macrophage infected with amastigote forms of *Leishmania* spp. (C). Positive correlation between IgG anti‐*Leishmania* and *Leishmania* spp. DNA (*r* = 0.87, *p* < 0.0001).

The cats in the leishmaniasis group showed no clinical signs consistent with those expected for the disease, such as skin lesions, lymph node enlargement, ophthalmic lesions, oral cavity lesions, weight loss, anorexia, or lethargy [2]. They also showed no abnormalities on general clinical examination and were considered asymptomatic. The detection of *Leishmania* spp. DNA in the bone marrow using the qPCR technique was positive, and the parasite load was measured in the cats (Figure 1A). IgG anti‐Leishmania antibodies were detected in the 21 cats in the leishmaniasis group, with an optical density of over 0.370; the control group showed no IgG reactivity to the total *L. infantum* antigen (Figure 1B). Intracellular amastigote forms of *Leishmania* were identified within bone marrow macrophages in infected cats. A representative photomicrograph from the infected group is shown in Figure 1C. A strong positive correlation between IgG anti‐*Leishmania* antibodies and *Leishmania* spp. DNA was observed (*p* < 0.0001) (Figure 1D).

In our study, two cats were co‐infected with FeLV and FelLV, and one cat with FIV and FelLV.

### 3.2 IL‐12 and IFN‐γ Were Increased in the Serum of Cats With Leishmaniasis

**FIGURE 2:**
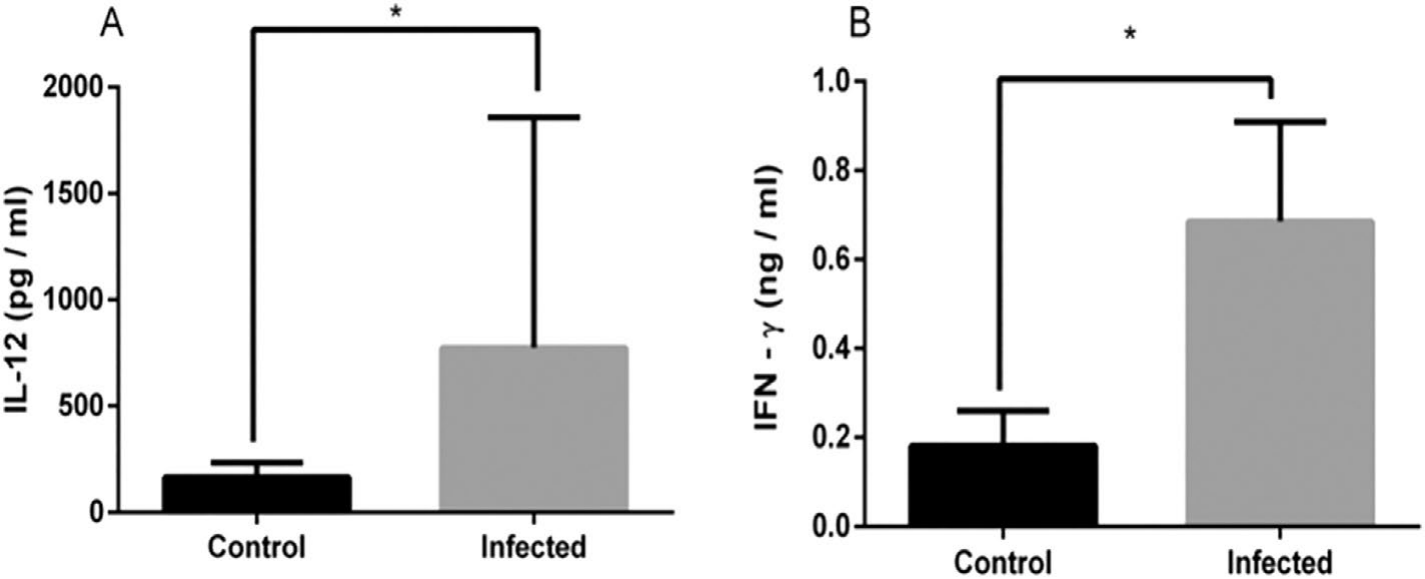
IL‐12 (A) and IFN‐γ (B) in serum from felines with leishmaniasis and healthy cats. Dual Ab sandwich ELISA was used to measure serum cytokines. Data presented with mean and standard error of the mean. Asterisk indicates a significant difference between the groups tested (*p* < 0.05).

Cats from an endemic area for visceral leishmaniasis produce IFN‐γ in vitro after stimulation of blood cells with soluble *Leishmania* spp. antigen [25] suggesting a cell‐type immune response in vivo. Thus, IL‐12 and IFN‐γ, cytokines of cellular immunity, were quantified in the serum of cats infected with *Leishmania* spp. IL‐12 (*p* = 0.0009) (Figure 2A) and IFN‐γ (*p* = 0.0487) (Figure 2B) were significantly elevated in cats with leishmaniasis compared to healthy cats.

### 3.3 IL‐12 Showed a Negative Correlation With Parasite Load and a Positive Correlation With IFN‐γ in Cats With Leishmaniasis

**FIGURE 3:**
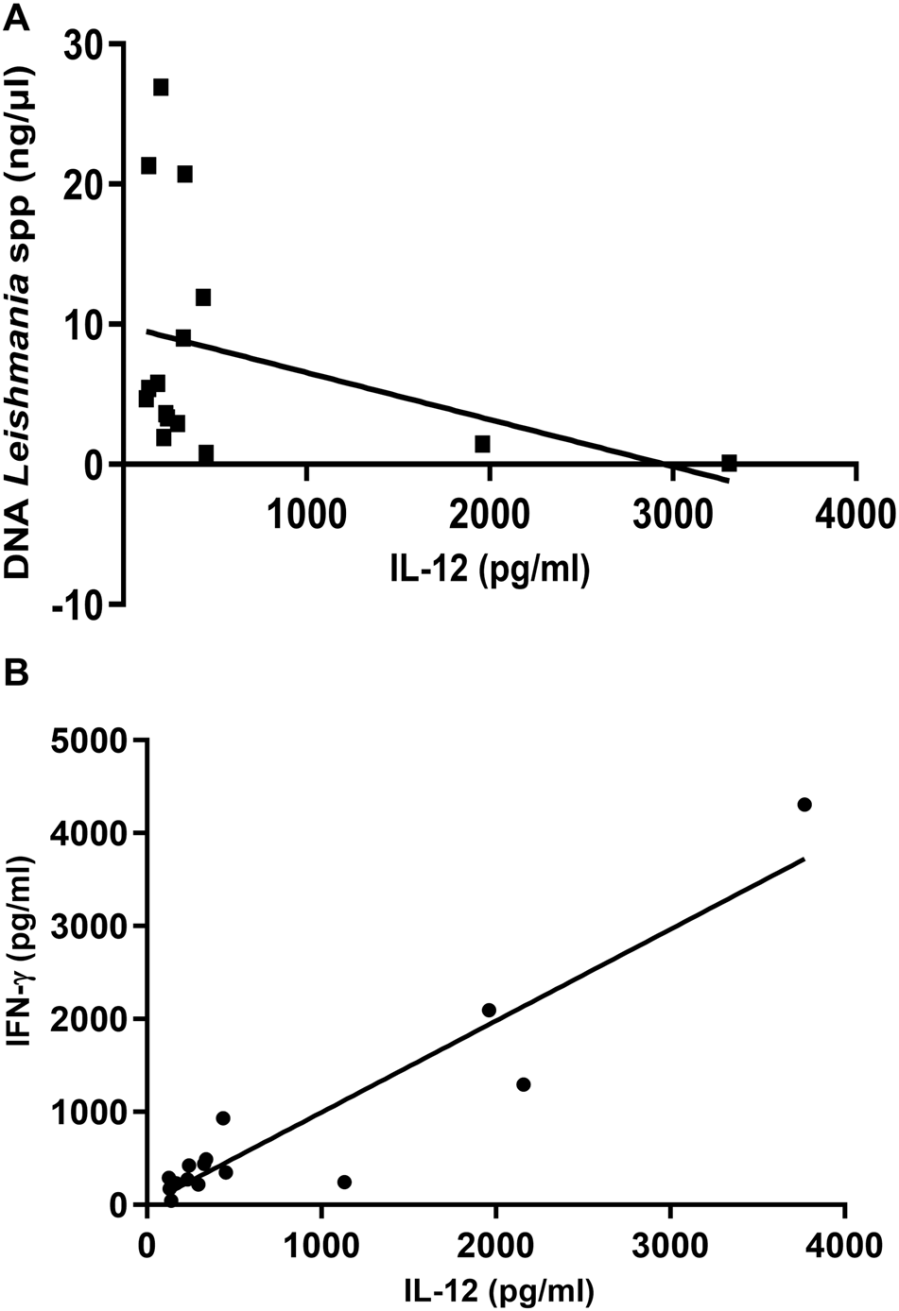
Negative correlation between serum IL‐12 levels and parasite load (*r* = −0.47, *p* = 0.036) (A) Positive association between levels of IFN‐γ and IL‐12 serum from infected cats (*r* = 0.87, *p* = 0.0007) (B).

The cytokine IL‐12 stimulates TCD4+ cells to secrete IFN‐γ to activate macrophages in order to eliminate the intracellular protozoa [23]. Therefore, we evaluated whether IL‐12 could regulate mechanisms associated with the control of parasite load in bone marrow in cats with leishmaniasis. We observed a negative correlation between serum IL‐12 levels and parasite load (*r* = −0.47, *p* = 0.036) (Figure 3A).

We also assessed the modulation of IL‐12 on IFN‐γ production. We observed a positive correlation between serum IL‐12 and IFN‐γ levels in cats with leishmaniasis (*r* = 0.87, *p* = 0.0007) (Figure 3B).

## 4 Discussion

Previous studies have shown the particularities of the immune response in different species to parasitic infection [30] and during leishmaniasis in dogs, there is an increase in the production of cytokines present in the Th2 response profile [20]. Considering the particularities of the species and the response profile during canine leishmaniasis, in this study we present that felines have the natural ability to mount a Th1‐skewed immune response during *Leishmania* spp. infection, favouring the intracellular elimination of parasites through an increase in IL‐12 and IFN‐γ, unlike what is observed during CVL.

In our study, the serum level of IL‐12 was high in cats with leishmaniasis without clinical signs of the disease. IL‐12 has an important role in the regulation of the cellular immune response in human leishmaniasis. IL‐12 restores cytotoxic function and IFN‐γ production that are essential for control of leishmanial multiplication [31]. In CVL, IL‐12 is important for the development of Th1 cells during experimental infection [32]. In cats, IL‐12 seems to be associated with controlling the clinical manifestations of FelVL, controlling parasitemia; future studies will be carried out to investigate the microbicidal mechanisms involved.

Consistent with the significant increase in IL‐12, IFN‐γ production was also significant in cats with leishmaniasis. A similar result was previously reported after blood cells from cats living in areas endemic for CVL and stimulated with *L. infantum* antigens produced IFN‐γ [25]. Dogs with mild or moderate leishmaniasis produce high levels of IFN‐γ and its absence is associated with the chronic stage [22]. Furthermore, an increase in IFN‐γ in dogs is associated with the absence of clinical signs of CVL [33]. Thus, it suggests that cats with asymptomatic leishmaniasis induce a cellular response with increased production of the cytokines IL‐12 and IFN‐γ, which contributes to a more effective response.

The negative correlation between parasite load in the bone marrow of cats and IL‐12 production suggests that IL‐12 may be contributing to parasite load control. IL‐12 is a pluripotent cytokine produced in the innate and adaptive immune response that interacts with NK and T cells to play a central role in the initiation and maintenance of Th1 responses and IFN‐γ production [34]. In mononuclear cells from patients with visceral leishmaniasis, IL‐12 exogenously augments IFN‐γ in response to *L. donovani* lysate [35]. In mononuclear cells from dogs with leishmaniasis, the cytokines rcaIL‐12/rcasIL‐10R1 induced IFN‐γ and TNF‐α production in PBMCs [33]. These results indicate that in cats, as in dogs and in humans, IL‐12 may play an important role in the regulation of cellular immune responses, helping to control parasitemia.

We observed a positive correlation between IL‐12 and IFN‐γ in the serum of cats infected with *Leishmania* spp. Similar results were observed in the culture supernatant of peripheral blood mononuclear cells from dogs, in which the cellular response was modulated via IFN‐γ after stimulation with IL‐12, inducing an increase in the production of NO [36], a molecule with microbicidal action against the parasite in CVL [37]. There are no studies on the action of NO during FelVL, but we suggest that due to the increased production of IFN‐γ, there is activation of infected macrophages with NO production. Future studies will be carried out to evaluate NO production in macrophages from cats with leishmaniasis.

Stimulation of cell differentiation towards a Th1 profile, associated with the release of IFN‐γ, may result in efficient control of parasite replication, contributing to a reduction in the clinical signs of the disease. Moreover, it should be noted that IL‐10 and TGF‐β levels are increased and associated with disease progression in human and canine visceral leishmaniasis [24, 38, 39]. Due to the experimental design of this study, it was not possible to measure Th2‐response cytokines in the serum of cats. It is essential that future studies further explore the interaction between the immune system and the expression of other cytokines involved in the disease in order to better clarify these mechanisms in cats.

## 5 Conclusion

We conclude that the interaction between IL‐12 and IFN‐γ plays a pivotal role in modulating the immune response in cats, aiding in the control of parasite replication and potentially preventing disease progression.

## Author Contributions

João Victor Lé Lode performed the experiments, acquisition of data, analysis, interpretation, and drafting of the manuscript. Lucas Takeshi Siqueira Ito and Gisele Mitsue Umino assisted in the experiments and analysed the data. Valéria Marçal Felix de Lima was responsible for project guidance, analysis, interpretation, and final drafting of the manuscript. All authors have read and approved the final manuscript.

## Conflicts of Interest

The authors declare no conflicts of interest.

## Peer Review

The peer review history for this article is available at https://www.webofscience.com/api/gateway/wos/peer‐review/10.1111/pim.70021.

## Data Availability

Data that supports the findings of this study are available upon request to the of bone marrow aspiration specimen corresponding author.
